# Phthalate Exposure from Drinking Water in Romanian Adolescents

**DOI:** 10.3390/ijerph15102109

**Published:** 2018-09-25

**Authors:** Rose O. Sulentic, Irina Dumitrascu, Nicole C. Deziel, Anca E. Gurzau

**Affiliations:** 1 Yale School of Public Health, Yale University, 60 College Street, New Haven, CT 06520, USA; rose.sulentic@yale.edu (R.O.S.); nicole.deziel@yale.edu (N.C.D.); 2 Environmental Health Center, Strada Busuiocului 58, 400240 Cluj-Napoca, Romania; irina.dumitrascu@ehc.ro

**Keywords:** adolescents, biomarkers, phthalates, Romania, water exposure

## Abstract

Phthalates are plastic softeners that have been linked to several adverse health outcomes. The relative contributions of different sources to phthalate exposure in populations in different regions and at different life stages is unclear. We examined the relationships between water consumption, consumer product use, and phthalate exposure among 40 adolescents (20 males, 20 females) in Cluj-Napoca, Romania. Interviewers administered a questionnaire about drinking water consumption and use of phthalate-containing consumer products. Four common phthalates were measured in representative samples of participants’ municipal drinking water and consumed bottled water using gas chromatography-mass spectrometry. Urine samples were collected from participants and analyzed for the corresponding phthalate metabolites. Relationships between different exposure measures were assessed using nonparametric tests (Spearman rank correlation coefficients and the Kruskal–Wallis test). Diisobutyl phthalate, dibutyl phthalate, and bis(2-ethylhexyl) phthalate were commonly detected in bottled water, but generally not the municipal drinking water samples. Mono-*n*-butyl phthalate (MnBP) was the most commonly detected urinary metabolite (detected in 92.5% of participants) and had the highest maximum concentration (1139.77 µg/g creatinine). We did not identify any statistically significant associations between water consumption or consumer product use practices and urinary phthalate metabolite concentrations in our adolescent group, and directions of correlation coefficients differed by individual phthalate compound. While phthalate exposure was widespread, these results highlight the challenges in examining phthalate exposure determinants and emphasize the need for further investigation into understanding exposure sources and potential health risks from chronic low-level exposures.

## 1. Introduction

Phthalates are plastic softeners found in a range of industrial and commercial products including flooring tiles, varnishes, food packaging materials, and personal-care products [1]. In addition, phthalates can be present in drinking water from contaminated source water or plastic pipes [2]. Consequently, human exposure may occur through multiple pathways and routes. Common phthalates include dibutyl phthalate (DBP), bis-2-ethylhexyl phthalate (DEHP), benzyl butyl phthalate (BBzP), diisobutyl phthalate (DiBP), and diethyl phthalate (DEP).

Phthalate exposure has been linked to several health outcomes across adult and child populations, the most well-studied being obesity. Associations with obesity have been reported in male children, male and female adolescents, and female adults [3]. Additionally, associations have been reported between exposure to DEP, DBP, and DEHP with increasing body mass index (BMI), waist circumference, and percent body fat in children [4]. Studies have observed endocrine-related effects such as low sperm count and poor sperm quality in adult males [5], and delayed pubic hair development in boys [6]. Associations have also been reported with DBP and increased risk of asthma [7]; monoethylhexyl phthalic acid (MEHP; a metabolite of DEHP) and insulin resistance, lower testosterone, and metabolic homeostasis [8]. The National Academy of Sciences lists phthalates as probable endocrine disruptors and carcinogens and emphasizes the need to identify the most important sources of phthalate exposure [9].

Exposure to phthalates in drinking water is an emerging area of public health concern. Studies suggest ingestion of water, as well as food, are important routes of exposure for phthalates [10,11]. Phthalates may be present in surface waters serving as municipal source waters due to industrial discharge or solid plastic waste; phthalates may also leach into municipal drinking water in distribution systems built with plastic high-density polyethylene (HDPE) or polyvinyl chloride (PVC) pipes. Tap water ingestion and absorption has been found to be a major source of exposure for DBP, DEHP, and di-*n*-octyl phthalate (DOP) in China [2]. Bottled water has been identified as a major source of phthalate intake in France [12].

Our study was conducted in the city of Cluj-Napoca, Romania where, like much of the European Union (EU), the municipal drinking water system is primarily composed of HDPE or PVC pipes. A prior study suggested phthalate concentrations in municipal drinking water may be of public health concern [13]. Romania is also an important place to conduct this research because there are no national or EU maximum admissible concentrations for phthalates in the public network or bottled drinking water. Additionally, Romania shares many characteristics of other southeastern European countries that might make the results of this study relevant to efforts across the EU. Studies examining phthalate exposure from plastic pipes are not only important for Romania and the EU’s existing systems, but have relevance to municipalities in the United States (U.S.) considering a transition from leaded to plastic pipes. Finally, Romania is also an interesting area to study because it recently experienced a rapid increase in urbanization, and consequently known phthalate-containing products (e.g., bottled beverages, personal care products, and packaged food) became more widely available.

Previous phthalate biomonitoring studies focused on pregnant or lactating women [11,14] and children [11,15] and suggest younger age groups have a higher exposure and vulnerability to phthalates than adults [16,17,18,19]. For example, a study that was part of a harmonized trans-European effort found that children had generally higher phthalate exposure than their mothers [20]. Other population subgroups have been observed to have relatively higher phthalate exposure and lower education and socioeconomic status being associated with increased phthalate metabolite concentrations [21]. Higher phthalate exposure has been observed amongst populations who engage in vigorous physical activity [22] and those using a higher number of different personal care products [23]. Therefore, this study focused on urban adolescents ages 12–17 in Cluj-Napoca, an entirely uninvestigated age group in Romania regarding phthalates.

The overarching goal of the study was to better understand the relationships between water consumption and consumer product use and phthalate exposure among adolescents in Romania. The specific objectives were (1) characterize drinking water consumption patterns of the adolescent population; (2) analyze tap and bottled water samples for the presences of phthalates; (3) evaluate the association between water consumption behaviors and the presence and concentrations of urinary phthalates metabolites; and (4) assess whether consumer product use was associated with phthalate biomarker concentrations.

## 2. Materials and Methods

### 2.1. Study Population

Study protocols were approved by the Human Investigation Committee at Yale University and the Environmental Health Center (Centrul de Mediu si Sanatate) in Cluj-Napoca, Romania. Potential participants were identified from the Environmental Health Center’s existing database of adult participants of previous drinking water studies who had consented to be recontacted regarding continuing or related drinking water studies. The adult participants were contacted by phone and asked whether they had a child between the ages of 12 and 17 currently resided in the cities of Cluj-Napoca or Floreşti. Adults with children of eligible ages were given further information on the study purpose and activities and asked permission to recruit their child. If permitted, we contacted their adolescent child by phone call to ascertain his or her interest and eligibility. Those who agreed were interviewed in their native language—Romanian or Hungarian—either at the subject’s home or the Environmental Health Center depending upon the subject’s preference. The study was approved by the Ethics Committee of Centrul de Mediu si Sanatate (registration number 290) on 6 April 2017 and Yale University (HIC #2000020986) on 19 May 2017.

### 2.2. Interview-Administered Questionnaire

We used a standardized, structured questionnaire to obtain information on demographics (e.g., participants education, parental/guardian education), health status (e.g., chronic disease history, weight, height) drinking water behavior at home and at school (e.g., satisfaction, consumption temperature, drinking vessel choice, storage choices, cooking use, household use), and other known phthalate sources from the subject’s consumer product use (e.g., beverages in plastic containers, microwaving food in plastic containers, food storage in plastic packaging, synthetic clothes, personal care products). Questions were designed to understand the magnitude and frequency of contact with phthalate-containing consumer products, particularly personal care products. We queried about several personal care products (shampoo, conditioner, body lotion or moisturizer, deodorant, shower gel, make-up, nail polish, perfume stored in plastic containers, toothpaste, medication, or nutritional supplements) and created a score with three categories: low (3–5 personal care products used weekly), medium (5–7 used weekly), and high (8–9 used weekly) for analysis.

For those reporting bottled water use, we queried brand names and consumption frequency of the bottled water both in general and specifically within the 24 h prior to the urine collection. We also asked whether the water was uncarbonated (“still”) or carbonated (“gas”) and queried the storage conditions (e.g., refrigerated, room temperature, in sunlight, and not in sunlight) for the bottled water for both general and previous 24-h consumption. Respondents identified their main reason for using bottled or tap water, and their opinions of the drinking water.

### 2.3. Collection of Tap Water

For participants reporting tap water as their primary drinking water source within the 24 h before the home visit (*n* = 33), we collected a 1-L sample either from their kitchen tap or, if a study participant served by the same public water system had already provided a sample, the previously collected sample was used, and no new sample was collected. In total, we collected 10 tap water samples, which were used to represent tap water for all participants primarily consuming tap water. Samples were collected in precleaned glass containers, transported back to the lab on ice, and extracted upon arrival.

### 2.4. Collection of Bottled Water

Participants with any bottled water use within the previous 24 h (*n* = 27) reported 16 unique combinations of brand, type (still or gas), and storage conditions (room temperature or refrigerated). Subsequently, we analyzed one sample for each of these 16 conditions (*n* = 16). We purchased the corresponding brands and recreated the reported storage conditions in the Environmental Health Center lab prior to analysis, to best represent the water consumed by participants. In the laboratory refrigerator, unopened in their original containers, we stored both gas (*n* = 3) and still (*n* = 5) samples at 1 to 4 degrees Celsius. We also stored the both gas (*n* = 5) and still (*n* = 3) samples at room temperature in a cupboard not in direct sunlight. No one reported storage conditions in direct sunlight in the previous 24 h of consumption, so we did not create these storage conditions.

### 2.5. Collection of Urine Samples

Prior to collection, urine specimen containers were assessed for the potential for phthalate leaching. Sample containers were filled with ultrapure water, stored in similar conditions as the samples, and then the water was analyzed for phthalate metabolites. All results were below detection limits.

At the time of interview, we provided participants with a urine collection container and asked them to collect their first morning void. They were asked to store the sample in cool conditions, out of direct sunlight and preferably refrigerated. Within 24 h of the void, we either transported the urine sample from the participants’ homes in a cooler or participants brought the samples to the lab. The samples were immediately frozen until laboratory analysis was possible.

### 2.6. Analysis of Phthalates in Tap and Bottle Water Samples

We analyzed all water samples for DiBP, DBP, BBzP, and DEHP. Sample analysis was performed according the ISO 18856, 2006 method. Compounds were extracted from water by solid phase extraction and identified and quantified using gas chromatography and mass spectrometry (GC-MS). The extraction cartridges were preconditioned by first washing with one column volume of ethyl acetate, vacuuming for 10 s, then washing with two column volumes of methanol. We then passed 250 mL samples through the column and vacuum dried the cartridge. The compounds were eluted with ethyl acetate. We transferred the samples into 1.5 mL vials, covered the vials with aluminum foil, closed the vial cap, and analyzed with GC-MS.

### 2.7. Phthalates Metabolite Analysis

We analyzed all urine samples for the urine metabolites corresponding to the parent phthalate compounds measured in water, namely MnBP, MEHP, MBzP, MEOHP, and MEHHP, according to Kim et al., 2014 [24], with minor modifications. A 2 mL aliquot of each urine sample was transferred into a borosilicate glass tube with ammonium acetate, and homogenized by vortexing. An internal standard and β-glucuronidase enzyme were added to each sample to deconjugate the metabolites of glucuronidated phthalates. The samples were covered with aluminum foil, washed with acetone and dried, gently agitated, and incubated at 37 °C in a closed water bath for 2 h. After enzymatic hydrolysis, 5 mL of extraction solvent were added to each sample and sonicated for 30 min. The aqueous phase was removed with a Pasteur pipette, and anhydrous sodium sulfate was added. The organic phase was transferred to an evaporation vial and evaporated to dryness under a low stream of nitrogen at 45 °C. A total of 100 μL of the derivatization agent *N*,*O*-bis (trimethylsilyl) trifluoracetamide with 1% trimethylchlorosilane (BSTFA with 1% TMCS) and 0.9 mL of ethyl acetate were added to each sample. The vials were covered with aluminum foil and placed in thermoreactor at 65 °C for 1 h. The samples were transferred into 1.5 mL vials and coated with aluminum foil before placing the vial cap and ultimately analyzed with GC-MS.

To correct for urine dilution, all samples were analyzed for creatinine. Samples were centrifuged for 15 min at 1600× *g*. A total of 1-mL of urine was transferred to a 100 mL volumetric flask and filled it to its nominal value with distillated water. A 2-mL aliquot of the diluted sample was transferred into a new vial, with 6 mL of picric acid and 0.4 mL sodium hydroxide (10%) added. After 20 min at room temperature, the sample was analyzed for creatinine by molecular spectrophotometry.

### 2.8. Quality Control

A blank and a laboratory control sample was analyzed for every 10 water or urine samples analyzed. Phthalates were not detected in any of the blank samples, indicating that no phthalate contamination occurred during sample analysis. The values for the control were all confirmed to be between the limits of a Shewhart Control Chart.

### 2.9. Statistical Analysis

We conducted descriptive statistics to characterize the drinking water and consumer product use in the study population. We also calculated summary statistics to describe the distribution of phthalate concentrations in water and urine samples. Samples with phthalate concentrations below the detection limit were assigned a value corresponding to half the limit of detection.

We estimated exposure to each phthalate compound occurring in the past 24 h from drinking water using the following equation.

$$\begin{array}{ll} \mathrm{Exposure}\;\left(µg\right)= & \left(\mathrm{Liters}\;\mathrm{of}\;\mathrm{bottled}\;\mathrm{water}\;\mathrm{consumed}\;\mathrm{in}\;\mathrm{past}\;24\;h\right)\\ & *\left(\mathrm{Phthalate}\;\mathrm{concentration}\;µg/L\right)\\ & +\left(\mathrm{Liters}\;\mathrm{of}\;\mathrm{tap}\;\mathrm{water}\;\mathrm{consumed}\;\mathrm{in}\;\mathrm{past}\;24\;h\right)\\ & *\left(\mathrm{Corresponding}\;\mathrm{phthalate}\;\mathrm{concentration}\;µg/L\right). \end{array}$$

This exposure variable was calculated separately for each of the phthalates. We evaluated associations between the calculated exposure to the parent phthalate in water and the corresponding urinary metabolite concentrations with Spearman rank correlation coefficients. We stratified the urinary metabolites by water consumption and consumer product usage of the subjects, and compared these ranges using the Kruskal–Wallis test.

## 3. Results

### 3.1. Drinking Water Patterns of Study Population

We recruited 20 female and 20 male adolescents for this study. The mean age of the participants was 15 years (SD = 1.8) and the average body mass index was 20.89 (SD = 3.8). Mirroring their age, participants were most likely to have 8 to 9 years of education (48%), followed by 5 to 7 years (27%), and 10 to 11 years (25%). The participant’s parents were most likely to have 15 to 19 years of education (55% mothers, 45% fathers), followed by 8 to 14 years (30% mothers, 37% fathers), and 20 to 25 years (15% mothers, 18% fathers).

### 3.2. Bottled and Tap Water Samples Phthalate Analysis

Phthalates were generally not detected in the tap water samples (Table 1), with only two samples having detectable concentrations of DiBP (0.084 and 0.104 µg/L). In contrast, several phthalates were detected in bottled water samples. Across all bottled water samples, the highest median concentration was observed for DBP, followed by DEHP and DiBP. Still water had higher median concentrations of DiBP and DBP compared to gas water, but lower median DEHP concentrations. Bottled water stored at room temperature had higher median concentrations of DiBP and DBP but lower median DEHP concentrations compared to bottled water stored refrigerated (Table A1 in Appendix A). BzBP was not detected in any tap or bottled water samples, and therefore was eliminated from further analysis.

### 3.3. Phthalate Metabolite and Water Consumption Analysis

Phthalate exposure was prevalent: all but two participants had detectable levels of at least one phthalate metabolite (Table 2). Percent detection varied widely across metabolites. MnBP was detected in 92.5% of samples, with median (IQR range) of 75.16 (34.70, 532.01) µg/L and 54.64 (24.02, 1139.77) when adjusted for creatinine (µg/g cr) (Table 2). Less than 50% of samples had detectable concentrations of MEHP, MEOHP, and MEHHP. MBzP was detected in 60% of samples, but the parent compound BzBP was not detected in any water samples. We restricted additional analyses to MnBP and MBzP, which had greater than 50% detection.

Calculated exposure to parent phthalates from drinking water based on Equation (1) yielded the following median and interquartile ranges. DBP exposure = 5.12 µg (3.12 µg, 7.66 µg); DEHP = 2.37 µg (0.22 µg, 5.02 µg); and DiBP = 1.67 µg (0.32 µg, 4.51 µg). Water exposure estimates were not correlated with urinary concentrations of corresponding metabolites (all Spearman correlation coefficients <0.125). DBP, the parent compound of MnBP, was most highly detected in the water samples, but we did not observe a correlation between them.

Table 3 presents the metabolite detection percentage and creatinine-adjusted concentrations by participant water consumption behaviors. While 98% of participants reported drinking some bottled water, approximately two-thirds (68%) of participants reported using tap water as their primary drinking water source at home; while at school, equivalent proportions of students reported drinking tap (43%) and bottled water (45%) as their primary source. Most (76%) reported being satisfied with the conditions of their chosen drinking water. The majority of participants reported consuming beverages other than water—like juice or soda—in plastic containers (83%). Use of bottled water for cooking was not common (3%); all (100%) reported tap water for other household purposes like laundry, hand-washing, or showering. Most then reported consuming tap water at the temperature running from the faucet (65%). Of those who drank bottled water, participants were more likely to drink still (65%) than gas (33%). Before consumption, most did not store the bottled water in a refrigerator (75%). Many stored water in plastic containers (43%). If consuming tap water, they typically used a glass vessel (73%). None of the water nor other beverages in plastic bottles consumption behaviors were statistically significantly associated with phthalate metabolite concentrations. Creatinine-adjusted MBzP concentrations were lower for those who stored bottled water in a refrigerator from those who did not store in a refrigerator, but the difference was not statistically significant (*p* < 0.1).

Table 4 describes metabolite detection percentage and creatinine-adjusted concentrations by consumer product use and demographics. Frequencies of individual personal product behaviors used to determine the personal care product use score are provided in Table A2, with the most common products being daily use of shampoo (100%), toothpaste (100%), and deodorant (89%). Far fewer participants reported the use of body lotion (*n* = 15), conditioner (*n* = 13), and make-up (*n* = 11), though of these individuals, some reported use of make-up (55%) and body lotion (53%) daily. Neither consumer product use nor demographics (age, sex, and BMI) were associated with phthalate metabolite concentrations. Those in the highest category of personal care product use had the highest concentrations of phthalate metabolites, but this relationship was not statistically significant.

## 4. Discussion

Our study used questionnaires, water samples, and urine phthalate metabolite concentrations to examine if water consumption influences phthalate exposure in adolescents. Most municipal water samples had no detectable levels of phthalates, while phthalates were commonly detected in bottled water samples. Phthalate metabolites were detected in 38 of the 40 participants urine samples. We did not find a significant association between drinking water phthalate exposure, consumer product usage, and urinary metabolite concentrations.

While we observed concentrations of DiBP of 0.084 and 0.104 µg/L in two of the municipal water samples collected in Cluj-Napoca and no detectable levels of DBP or DEHP, a previous study found 0.05 µg/L DBP in one sample in Dâmbu Rotund and values up to 0.3 µg/L DiBP in the water throughout Florești, Gruia, and Dâmbu Rotund areas of Cluj-Napoca [13]. There may have been temporal, temperature, site, and infrastructure discontinuities that may explain the differences between Dumitrascu results and ours. We sampled during the summer months of June and July 2017, while Dumitrascu sampled during fall in September 2014 and winter in February 2015. The two studies collected water at different locations within the water distribution systems. Lastly, several years elapsed between our collection and their study. The pipe infrastructure could have changed through degradation or construction in that time.

Bottled water storage conditions may play an important role in the presence and concentrations of phthalates in drinking water. Previous studies have demonstrated that bottle water samples stored at higher temperatures have higher concentrations of phthalates [25,26]. Consistent with the trend in the literature, we observed higher median concentrations of DiBP and DBP in room temperature samples compared to those which were refrigerated (Table A1). In contrast, in our recreated storage conditions, bottled water stored at room temperature had lower median concentrations of DEHP than those stored in a refrigerator; however, this unexpected finding was consistent with Al-Saleh et al., 201l [25], who observed significantly lower DEHP levels in ten brands of bottled water stored at room temperature compared to those stored at 4 °C in Saudi Arabia. We observed a suggestion of elevated median MnBP concentrations among those who typically consumed bottled water stored at room temperature (median value = 54.6 µg/g creatinine) versus in a refrigerator (median value = 20.3 µg/g creatinine) (Table 3). The higher observed concentrations of DBP in bottled water stored at room temperature could be influencing the higher MnBP concentrations in urine. We also observed some differences in concentrations across brands, still versus gas, and storage temperature (Table A1). However, the assessment of storage conditions in our study was designed to recreate the phthalate exposure during the time period corresponding to the biological monitoring, and not provide a systematic analysis of the impact of storage conditions on phthalate concentrations. Therefore, it is possible these observations are due to between-bottle variability or differences in the type of plastic used between unique brands. 

We contextualized water consumption behaviors with other consumer product use linked to phthalate exposure (Table 4). We observed a higher median and interquartile range of urinary MnBP concentration among those who use plastic wrap for food storage (median value = 67.8 µg/g creatinine) and those who do not (median value = 39.5 µg/g creatinine) (*p* < 0.10). The elevated levels in those who use plastic wrap for food storage is consistent with the evidence suggesting phthalates can migrate from plastic food storage material into the food, and potentially ingested [27]. However, we also observed an inverse relationship in the interquartile range of MBzP between those who use plastic packaging for food storage (median value = 15.2 µg/g creatinine) and those who do not (median value = 22.4 µg/g creatinine) (*p* < 0.10) even though MBzP’s parent compound BzBP can migrate from food storage materials and has been detected in food products [28].

There were several behaviors we might have expected to see an increasing correlation with phthalate metabolite concentrations and did not. Such behaviors include microwaving food in plastic containers [29] and increasing personal care product use [23]. While we did observe a higher metabolite concentration in the group that used the most personal care products, such measurements overlapped with the interquartile range and were subsequently not significant. The lack of correlation may be reflective of our limited study size. It is possible we did not get a complete random sampling adequate to capture the relationship between metabolites and the potential exposures from behaviors. Alternatively, it is possible that other sources, such as consumption of food or inhalation of air, are greater contributors to exposure in this population. Also, the timing of reported activities (e.g., microwaving in plastic) reflects usual behaviors and may not have been representative of behaviors corresponding to the timing of the urine collection.

The phthalate concentrations observe in our adolescent participants were in a similar range of values reported in other populations, though differences could be observed depending on the compound and the population. The median concentration of MnBP (24 µg/g creatine) was higher than that observed in Austrian children ages 7 to 15 (12 µg/g creatinine) [18] and similar to that found in Flemish adolescents ages 14 to 15 (28.44 µg/g creatine) [30]. We observed a median concentration of MBzP (19 µg/g creatinine), higher than Austrian children ages 7 to 15 (2.5 µg/g creatinine) [19], but lower than Flemish adolescents ages 14 to 15 (22.45 µg/g creatine) [30]. Our population had a median concentration of MEHP below the detection limit, while Austrian children ages 7 to 15 had a median concentration of 0.94 µg/g creatinine [18] and Flemish adolescents ages 14 to 15 had a median concentration of 2.67 µg/g creatine [30]. In our study, both MEOHP and MEHHP had median concentrations below the detection limit, while Austrian children ages 7 to 15 had median concentrations of 31 µg/g creatinine and 3.3 µg/g creatinine [18], and Flemish adolescents ages 14 to 15 had a median concentration of 19.95 µg/g creatine and 15.06 µg/g creatine [30], respectively.

Our adolescents had a higher geometric mean concentration of MnBP (49.57 µg/g creatinine) compared to Korean adolescents (33.14 µg/g creatinine) [19]. The adolescents in our study also had higher concentrations of MBzP (geometric mean of 10.65 µg/g creatinine) than Korean adolescents (4.28 µg/g creatinine) [19]. Our observed geometric mean for MEOHP was 2.59 µg/g creatinine, lower than Korean adolescents (12.26 µg/g creatinine) [19]. Similarly, our participants geometric mean for MEHHP was 1.48 µg/g creatinine, lower than that observed in a study of Korean adolescents (17.03 µg/g creatinine) [19]. The values for MEPH metabolites in Korean adolescents are not available for comparison.

Previous studies conclude younger age groups are more vulnerable and have higher exposure to phthalates compared to adults [16,17,18,19]. In comparisons with adults, our adolescent participants had a geometric mean of MEHP (2.00 µg/g creatinine), lower than mothers of the Czech Republic (3.13 µg/g creatinine), Slovakia (3.14 µg/g creatinine), Hungary (3.70 µg/g creatinine) [31], and Ireland (2.8 µg/g creatinine) [32]. The adolescents in our study had higher concentrations of MBzP (geometric mean of 10.66 µg/g creatinine) compared to the maternal populations in Czech Republic (4.35 µg/g creatinine), Slovakia (3.81 µg/g creatinine), Hungary (3.82 µg/g creatinine) [31], and Ireland (3.1 µg/g creatinine) [32]. Certain phthalate metabolites (MEOHP, MEHHP, and MnBP) were lower in our study population, compared to previous European maternal studies. Our geometric mean for MEOHP was 2.59 µg/g creatinine, much lower than Czech Republic (11.68 µg/g creatinine), Slovakia (11.54 µg/g creatinine), Hungary (10.37 µg/g creatinine) [31], and Ireland (8.8 µg/g creatinine) [32]. Our participants geometric mean for MEHHP was 1.48 µg/g creatinine, lower compared to Czech Republic (18.45 µg/g creatinine), Slovakia (18.20 µg/g creatinine), Hungary (15.54 µg/g creatinine) [31], and Ireland (17.0 µg/g creatinine) [32]. MnBP was not reported in Cerna et al., 2015 [31] for comparison, but was included in Cullen et al., 2017 [32]. Irish mothers had a geometric mean of 23.8 µg MnBP/g creatinine [32], much lower than our adolescents 49.57 µg/g creatinine.

While it appears that our adolescents generally had comparable or lower concentrations of phthalate metabolites compared to other studies of European adults, differences may be related to a small sample size or intercountry differences in exposures, or differences in urine collection methods (e.g., first morning void versus 24-h sample). We would need additional data to fully understand Romanian adolescent and adult exposure and provide further context for comparison.

Limitations of this study include the low percent detection of phthalates metabolites in urine, and the sample size of 40, both of which limited our power and statistical analysis. Additionally, in our bottled water analyses, we recreated water storage conditions, instead of using a sample of the actual bottled water participants consumed. To address this limitation, we matched the brand and storage conditions to the best of our abilities. Another limitation is the use of morning first urine sample instead of 24 h urine sample.

One of the strengths of this study is the use of urine metabolite analysis, consistent with methods promoted by DEMOCOPHES studies, facilitating comparisons with other populations [31,32,33]. It is difficult to compare the results of our study to that of DEMOCOPHES because adolescent results are limited. However, the urinary concentrations of some metabolites (MEHP, MBzP, MEOHP, MEHHP, and MnBP) were comparable to other adolescent studies [18,19].

Another strength of this study includes the examination of an understudied group—adolescents. Adolescents are especially important to understand plasticizer exposure because of their endocrine system vulnerability. The endocrine system becomes increasingly active throughout puberty until operating at full maturity [34]. Disruptions to this maturing system during childhood or adolescence will have ramifications throughout the rest of the individual’s lifetime. Additionally, this population reported a high prevalence of bottled drink consumption, making them a particularly important group for this area of study. Finally, adolescents are a particularly important group for understanding ways to curb plasticizer exposure because of their potential for education and adaptation.

## 5. Conclusions

Phthalate exposure was nearly ubiquitous in our study population; phthalates were detected in bottled water samples, but generally not municipal water. We did not identify water consumption or consumer product use as major sources contributing to phthalate exposure. Further investigation into the contribution of different sources to overall exposure could help inform exposure mitigation strategies across different population groups and life stages. Further studies should examine whether this low-level but potentially continuous exposure from multiple sources could pose a potential health threat.

## Figures and Tables

**Table 1:** Concentrations of phthalates in tap and bottled water samples.

			Phthalate Concentrations (µg/L) Median (IQR)		
		*N*	DiBP	DBP	DEHP
Tap (*n* = 10)	All	10	ND (ND, ND)	ND (ND, ND)	ND (ND, ND)
Bottled (*n* = 16)	All	16	0.77 (0.25, 2.50)	3.23 (ND, 6.15)	2.18 (0.31, 4.97)
	Still water	8	1.36 (0.25, 5.16)	5.61 (1.56, 8.44)	1.26 (0.11, 3.70)
	Gas water	8	0.48 (0.22, 2.23)	2.16 (ND, 3.87)	3.14 (0.67, 6.50)
	Room temperature	8	2.23 (0.38, 3.61)	3.87 (ND, 6.15)	1.45 (0.20, 3.98)
	Refrigerated	8	0.41 (0.17, 1.36)	3.05 (0.68, 6.39)	2.61(0.58, 5.25)

Detection limit is 0.015 µg/L. ND signifies below detection limit. BzBP had no samples with detectable concentrations. DiBP—diisobutyl phthalate; DBP—dibutyl phthalate; BzBP—Benzylbutyl phthalate; DEHP—Bis(2-ethylhexyl) phthalate; IQR, interquartile range.

**Table 2:** Concentrations of phthalate creatinine adjusted metabolites (µg/g cr) in urine samples.

Adjusted Metabolite (µg/g cr)	*N* Detected	% Detected	Geometric Mean	Min	25th Percentile	Median	75th Percentile	Max
MnBP	37	92.5	142.32	ND	24.02	54.64	105.56	1139.77
MEHP	16	40.0	3.14	ND	ND	ND	2.52	16.08
MBzP	24	60.0	27.21	ND	ND	19.00	38.97	139.90
MEOHP	3	7.5	1.85	ND	ND	ND	3.76	5.84
MEHHP	13	32.5	10.08	ND	ND	ND	4.38	152.40

Detection limit = 2.5 µg/L. ND signifies below detection limit. MnBP—Mono-*n*-butyl phthalate; Monoethylhexyl phthalic acid; MBzP—Mono-benzyl phthalate; MEOHP—Mono(2-ethyl-5-oxohexyl)phthalate; MEHHP—Mono(2-ethyl-5-hydroxyhexyl)phthalate.

**Table 3:** Metabolite detection and creatinine-adjusted concentrations (µg/g creatinine) by water consumption behavior.

Water Consumption Behavior	*N* (%)	Med (IQR)		Detection *N* (%)				
		MnBP ^a^	MBzP ^a^	MnBP ^b^	MEHP ^b^	MBzP ^b^	MEOHP ^b^	MEHHP ^b^
Main source of drinking water at home								
Tap water	27 (68)	66.9 (25.7, 141.7)	19.2 (2.2, 55.1)	27 (100) **	12 (44)	17 (63)	2 (7)	10 (37)
Bottled water	13 (32)	35.7 (18.2, 96.4)	15.3 (1.9, 23.4)	10 (77) **	4 (31)	7 (54)	1 (8)	3 (23)
Main source of drinking water at school								
Tap water (fountains)	17 (43)	59.9 (25.7, 96.4)	16.2 (1.9, 20.7)	16 (94)	7 (41)	10 (59)	2 (12)	6 (35)
Bottled water	18 (45)	54.6 (20.3, 114.0)	24.7 (2.7, 62.2)	16 (89)	8 (44)	12 (67)	1 (6)	7 (39)
Missing/Do not know	5 (13)	37.6 (24.5, 95.0)	20.7 (2.2, 48.7)	5 (100)	1 (20)	3 (60)	0 (0)	0 (0)
Opinion of drinking water taste								
Satisfied	31 (76)	49.6 (23.5, 96.4)	19.2 (2.5, 48.7)	29 (94)	14 (45)	20 (65)	2 (6)	11 (35)
Dissatisfied	4 (10)	105.6 (50.0, 154.0)	22.9 (11.8, 35.1)	3 (75)	2 (50)	3 (75)	0 (0)	2 (50)
No opinion	5 (13)	37.6 (35.7, 95.0)	2.1 (1.3, 2.2)	5 (100)	0 (0)	1 (20)	1 (20)	0 (0)
Drinks beverages (other than water) in plastic containers								
Yes	33 (83)	49.6 (20.3, 114.0)	19.2 (ND, 41.5)	30 (91)	13 (40)	20 (61)	3 (9)	10 (30)
No	7 (17)	59.7 (37.6, 97.1)	15.2 (2.12, 22.4)	7 (100)	3 (43)	4 (57)	0 (0)	3 (43)
Main source of water for cooking								
Tap water	37 (93)	59.7 (23.5, 114.0)	19.2 (2.2, 41.5)	34 (92)	15 (41)	23 (62)	3 (8)	13 (35)
Bottle water	3 (8)	35.7 (28.8, 59.9)	1.9 (ND, 28.6)	3 (100)	1 (33)	1 (33)	0 (0)	0 (0)
Tap water as main source for household purposes (laundry, hand-washing, showering)								
Yes	40 (100)	54.6 (24.0, 105.6)	19.0 (2.1, 39.0)	37 (93)	16 (40)	24 (60)	3 (8)	13 (33)
No	0 (0)							
Usual temperature of water consumed								
Temperature as running from faucet	26 (65)	44.6 (23.5, 120.1)	21.5 (2.7, 48.7)	23 (88)	12 (46)	18 (69)	1 (4)	9 (35) **
Room temperature	4 (10)	44.3 (20.9, 77.4)	2.4 (1.8, 28.8)	4 (100)	1 (25)	1 (25)	0 (0)	0 (0) **
Refrigerated	9 (23)	35.7 (20.3, 67.8)	4.9 (1.9, 16.2)	9 (100)	2 (22)	4 (44)	2 (22)	3 (33) **
Missing	1 (3)							
Drinks bottled water								
Still	26 (65)	39.1 (18.2, 114.0)	22.4 (2.5, 46.8)	23 (88)	11 (42)	18 (69)	1 (4)	10 (39)
Gas	13 (33)	67.8 (26.9, 97.1)	16.2 (ND, 36.5)	12 (92)	6 (46)	7 (54)	2 (15)	4 (31)
None	1 (2)							
Usual storage conditions of bottled water consumed								
Unrefrigerated	30 (75)	54.6 (25.7, 114.0)	21.5 (2.2, 48.7)	27 (90)	13 (43)	19 (63)	2 (7)	12 (40)
Refrigerator	5 (13)	20.3 (18.2, 35.7)	15.3 (1.9, 28.6)	5 (100)	1 (20)	3 (60)	1 (20)	0 (0)
Missing/Do not know	5 (13)							
Usual vessel for tap water storage at room temperature								
Plastic vessel	17 (43)	66.9 (24.5, 95.0)	4.9 (ND, 28.6)	16 (94)	3 (18) **	8 (47)	1 (6)	4 (24)
Glass vessel	13 (32)	38.6 (6.5, 96.4)	20.7 (3.9, 36.5)	10 (77)	8 (62) **	9 (69)	0 (0)	4 (31)
Missing/Do not know	10 (25)							
Usual vessel for tap water consumption								
Glass	29 (73)	49.6 (23.5, 114.0)	20.7 (3.90, 48.72)	27 (93)	13 (45)	20 (69) **	2 (7)	10 (34)
Plastic	4 (10)	9.7 (4.6, 39.9)	2.6 (2.04, 11.67)	3 (75)	0 (0)	1 (25) **	0 (0)	0 (0)
Mug	4 (10)	31.3 (6.5, 51.7)	24.6 (18.42, 32.54)	4 (100)	1 (25)	4 (100) **	0 (0)	1 (25)
Missing/Other/Do not know	3 (7)							
	Mean (Std Dev)	MnBP ^c^	MBzP ^c^					
Amount of water consumed daily (liters)	1.4 (0.62)	−0.08	0.0					
Male participants	1.6 (0.78)	−0.3	0.1					
Female participants	1.2 (0.34)	0.1	0.0					
Amount of bottled water consumed daily (liters)	0.8 (0.65)	−0.4	0.1					
Amount of tap water consumed daily (liters)	1.0 (0.53)	0.3	−0.21					

^a^ = Kruskal–Wallis test; ^b^ = Chi square test; ^c^ = Spearman correlation; ** = *p* < 0.05. Detection limit is 2.5 µg/L. ND signifies below detection limit. MnBP—Mono-*n*-butyl phthalate; Monoethylhexyl phthalic acid; MBzP—Mono-benzyl phthalate; MEOHP—Mono(2-ethyl-5-oxohexyl)phthalate; MEHHP—Mono(2-ethyl-5-hydroxyhexyl)phthalate.

**Table 4:** Metabolite detection and creatinine-adjusted concentrations (µg/g creatinine) by consumer product use and demographics.

	*N* (%)	Med (IQR)		Detection *N* (%)				
		MnBP ^a^	MBzP ^a^	MnBP ^b^	MEHP ^b^	MBzP ^b^	MEOHP ^b^	MEHHP ^b^
Consumer product use and behavior								
Microwaves food in plastic containers								
Yes	4 (10)	90.5 (41.8, 147.8)	10.2 (ND, 32.79)	4 (100)	0 (0)	2 (50)	0 (0)	1 (25)
No	36 (90)	44.6 (6.46, 96.8)	19.6 (2.2, 39.0)	33 (92)	16 (44)	22 (61)	3 (8)	12 (33)
Plastic packaging for food storage								
Yes	17 (42)	38.6 (16.7, 70.7)	15.2 (ND, 22.4)	16 (94)	5 (29)	9 (53)	0 (0)	2 (12) **
No	23 (58)	67.8 (24.5, 141.7)	22.4 (2.7, 48.7)	21 (91)	11 (48)	15 (65)	3 (13)	11 (48) **
Plastic containers for food storage								
Yes	12 (30)	53.2 (14.5, 117.0)	19.7 (2.1, 38.8)	11 (92)	4 (33)	8 (67)	0 (0)	3 (25)
No	28 (70)	54.6 (25.1, 95.7)	17.3 (2.1, 39.0)	26 (93)	12 (43)	16 (57)	3 (11)	10 (36)
Plastic bags for food storage								
Yes	18 (45)	77.1 (37.6, 97.1)	15.7 (ND, 30.8)	18 (100)	6 (33)	10 (56)	1 (6)	6 (33)
No	22 (55)	34.5 (16.7, 120.1)	19.6 (2.5, 41.5)	19 (86)	10 (45)	14 (64)	2 (9)	7 (32)
Plastic wrap for food storage								
Yes	11 (28)	67.8 (37.6, 189.9)	3.9 (ND, 36.5)	11 (100)	4 (36)	5 (45)	1 (9)	5 (45)
No	29 (72)	39.5 (18.2, 96.4)	20.6 (2.5, 41.5)	26 (90)	12 (41)	19 (66)	2 (7)	8 (28)
Clothing								
Only cotton/natural	14 (35)	37.6 (25.7, 70.7)	12.5 (ND, 28.6)	12 (86)	6 (43)	7 (50)	1 (7)	5 (36)
Only synthetic	4 (10)	42.3 (11.6, 75.6)	19.7 (9.8, 28.6)	4 (100)	1 (25)	3 (75)	0 (0)	1 (25)
Both	21 (53)	95.0 (24.5, 181.5)	19.2 (2.7, 48.7)	20 (95)	9 (43)	14 (67)	2 (10)	7 (33)
Do not know	1 (3)							
Personal care product use								
Low	25 (63)	38.6 (23.5, 97.1)	18.8 (1.9, 28.6)	23 (92)	10 (40)	16 (64)	3 (12)	7 (28)
Medium	8 (20)	53.2 (20.8, 78.0)	12.8 (2.0, 62.1)	8 (100)	4 (50)	4 (50)	0 (0)	4 (50)
High	7 (17)	95.0 (37.6, 120.1)	22.4 (2.1, 55.1)	6 (86)	2 (29)	4 (57)	0 (0)	2 (29)
Demographic characteristic								
Age (years)								
12–15	20 (50)	54.8 (25.1, 143.5)	9.6 (1.8, 37.4)	17 (85)	8 (40)	10 (50)	2 (10)	8 (40)
16–18	20 (50)	48.6 (20.8, 105.2)	19.9 (3.7, 39.0)	20 (100)	8 (40)	14 (70)	1 (5)	5 (25)
Sex								
Male	20 (50)	60.1 (19.2, 161.6)	17.47 (ND, 24.7)	18 (90)	8 (40)	12 (60)	3 (15)	5 (25)
Female	20 (50)	49.6 (27.2, 91.7)	21.2 (2.17, 51.9)	19 (95)	8 (40)	12 (60)	0 (0)	8 (40)
	Mean (Std Dev)	MnBP ^c^	MBzP ^c^					
BMI	20.9 (3.8)	−0.1	0.0					

^a^ = Kruskal–Wallis test; ^b^ = Chi square test; ^c^ = Spearman correlation; ** = *p* < 0.05; Detection limit is 2.5 µg/L. ND signifies below detection limit. MnBP—Mono-*n*-butyl phthalate; Monoethylhexyl phthalic acid; MBzP—Mono-benzyl phthalate; MEOHP—Mono(2-ethyl-5-oxohexyl)phthalate; MEHP—Mono(2-ethyl-5-hydroxyhexyl)phthalate. Personal care product use categories: low—1 to 4 PCP used, 5 to 7 PCP used, 8 to 9 PCP used.

## Appendix A

**Table A1:** Concentrations of phthalates in bottled water by characteristic and storage condition.

	Room Temperature (*n* = 8)	Refrigerator (*n* = 8)
	**DiBP**	
Still water (*n* = 8)	4.51 (0.29, 9.14) *n* = 3	1.04 (0.22, 1.67) *n* = 5
Gas water (*n* = 8)	2.17 (0.47, 2.29) *n* = 5	0.32 (0.12, 0.50) *n* = 3
	**DBP**	
Still water (*n* = 8)	6.11 (ND, 17.79) *n* = 3	5.12 (3.12, 7.66) *n* = 5
Gas water (*n* = 8)	3.34 (ND, 6.20) *n* = 5	1.35 (ND, 2.97) *n* = 3
	**DEHP**	
Still water (*n* = 8)	0.52 (ND, 2.37) *n* = 3	2.00 (0.22, 5.02) *n* = 5
Gas water (*n* = 8)	3.06 (0.39, 4.91) *n* = 5	3.22 (0.94, 8.09) *n* = 3

**Table A2:** Personal-care product usage by participants.

	Any Use *N* = 40	Participant Frequency *N* (%)			
Product	*N*	1–2 Days/wk	3–5 Days/wk	6–7 Day/wk	Daily
Shampoo	40	18 (45)	15 (37)	1 (3)	6 (15)
Toothpaste	40	0 (0)	0 (0)	0 (0)	40 (100)
Deodorant	37	0 (0)	4 (11)	0 (0)	33 (89)
Shower gel	35	0 (0)	6 (17)	1 (3)	28 (80)
Body lotion/moisturizer	15	6 (40)	1 (7)	0 (0)	8 (53)
Conditioner	13	8 (62)	4 (31)	1 (7)	0 (0)
Make-up	11	2 (18)	2 (18)	1 (9)	6 (55)
Perfume stored in plastic containers	6	3 (50)	0 (0)	0 (0)	3 (50)
Nail polish	5	0 (0)	0 (0)	0 (0)	5 (100)
